# Creating and Validating a Questionnaire on Dentists’ Perceptions Regarding Periodontics–Prosthodontics Interdisciplinary Clinical Practice

**DOI:** 10.3390/clinpract15080149

**Published:** 2025-08-07

**Authors:** Gabriel Rotundu, Dragos Ioan Virvescu, Zinovia Surlari, Dana Gabriela Budala, Florin Razvan Curca, Carina Balcos, Cristian Cojocaru, Vlad Constantin, Razvan Gradinariu, Ionut Luchian

**Affiliations:** 1 Department of Periodontology, Faculty of Dental Medicine, “Grigore T. Popa” University of Medicine and Pharmacy, 700115 Iasi, Romania; 2 Department of Dental Materials, Faculty of Dental Medicine, “Grigore T. Popa” University of Medicine and Pharmacy, 700115 Iasi, Romania; 3 Department of Fixed Prosthodontics, Faculty of Dental Medicine, “Grigore T. Popa” University of Medicine and Pharmacy, 700115 Iasi, Romania; 4 Department of Complete Dentures, Faculty of Dental Medicine, “Grigore T. Popa” University of Medicine and Pharmacy, 700115 Iasi, Romania; 5 Department of Implantology and Removable Prosthodontics, Faculty of Dental Medicine, “Grigore T. Popa” University of Medicine and Pharmacy, 700115 Iasi, Romania; 6 Department of Oral Health, Faculty of Dental Medicine, “Grigore T. Popa” University of Medicine and Pharmacy, 700115 Iasi, Romania

**Keywords:** prosthetic restorations, periodontal health, dentists’ perceptions, questionnaire validation, exploratory factor analysis, internal consistency

## Abstract

**Background:** The interaction between prosthetic restorations and periodontal health is a critical factor for the long-term success of dental treatments. A biologically compatible prosthetic design supports periodontal stability, whereas neglecting periodontal principles can compromise treatment outcomes. This study aimed to validate a questionnaire designed to assess dentists’ perceptions regarding the influence of prosthetic restorations on the periodontium. **Material and Methods:** An observational cross-sectional study was conducted using a self-administered questionnaire distributed to licensed dentists across Romania. The questionnaire underwent expert review, pilot testing (*n* = 50), and statistical validation, including the Content Validity Index (CVI), Cronbach’s alpha for internal consistency, and Exploratory Factor Analysis (EFA) using Principal Component Analysis (PCA) with Varimax rotation. The final sample included 39 respondents. Data was analyzed using SPSS v26.0. **Results:** The questionnaire demonstrated excellent internal consistency (Cronbach’s alpha = 0.900; standardized alpha = 0.917). Most items had corrected item-total correlations > 0.40. EFA revealed eight coherent factors explaining 81.68% of total variance, with high communalities (0.549–0.966), strong Kaiser–Meyer–Olkin test (KMO) values, and significant Bartlett’s test values, confirming construct validity. Descriptive statistics showed predominantly positive attitudes among dentists regarding the periodontal considerations in prosthetic treatment. The highest-rated items emphasized oral hygiene, periodontal stability, and biological adaptation of restorations. Lower scores were associated with routine use of periodontal indices and recognition of failures due to insufficient evaluation. **Conclusions:** The validated instrument proved reliable and demonstrated strong psychometric properties in this exploratory validation, supporting its use in research and education. Romanian dentists demonstrated a favorable perception of the role of periodontal health in prosthetic success. This tool can inform curriculum development and interdisciplinary clinical protocols in prosthodontics and periodontology.

## 1. Introduction

The relationship between prosthetic restorations and periodontal health is a critical consideration in modern dental practice. A successful prosthetic treatment must go beyond functional and aesthetic outcomes to ensure compatibility with the periodontal tissues and long-term oral health. Inappropriate design, marginal misfit, or poor hygiene accessibility can all contribute to periodontal inflammation, tissue breakdown, and ultimately prosthetic failure [1,2].

In clinical practice, numerous factors related to prosthetic design and execution can have a significant impact on periodontal tissues [3,4]. These include marginal fit, emergence profile, contouring, occlusal loading, and the choice of materials, as well as the patient’s ability to maintain proper oral hygiene around the restoration [5,6]. Faulty prosthetic restauration or neglect of periodontal considerations may contribute to plaque accumulation, gingival inflammation, attachment loss, and eventual prosthesis failure. Conversely, a well-designed and biologically compatible prosthetic restoration can support periodontal health and even contribute to the rehabilitation of patients with previously compromised dentition [7,8,9].

Despite the clinical importance of the prosthesis–periodontium relationship, there is limited data on how general dentists perceive and incorporate these principles into daily practice [10]. The way dentists conceptualize the impact of prosthetic treatments on periodontal tissues can influence not only the quality of their work but also interprofessional collaboration with periodontists and hygienists [11]. Moreover, their level of awareness may reflect the strengths and weaknesses of academic training or continuing education in this area [12,13].

This study aims to validate a newly developed questionnaire designed to assess dentists’ perceptions regarding the influence of prosthetic restorations on the periodontium. The instrument was constructed to evaluate multiple dimensions, including knowledge of biological principles, perceived clinical priorities, decision-making in prosthetic design, and attitudes toward interdisciplinary collaboration. The validation process involves expert evaluation, pilot testing, and statistical analysis of reliability and internal consistency metrics.

Creating assessment instruments that are both valid and reliable is fundamental in healthcare research. Surveys and questionnaires are commonly employed to investigate the knowledge, attitudes, and practices of medical professionals [14]. Nevertheless, for the data they produce to be accurate and applicable to broader populations, these tools must be subjected to a thorough validation process [15]. This involves evaluating the relevance of the content, the clarity of the items, the internal consistency of responses, and the overall construct validity. In the absence of such validation, the findings obtained from questionnaire-based studies risk being inaccurate or lacking scientific credibility [16,17,18].

By providing a standardized and validated instrument, this research seeks to contribute to a deeper understanding of how dental professionals engage with the prosthetics–periodontium interface.

Such knowledge can inform future educational interventions, curriculum development, and quality improvement initiatives in prosthetic dentistry and periodontology. Ultimately, improving practitioners’ awareness and competence in this domain may lead to better clinical outcomes, enhanced patient satisfaction, and more sustainable oral health care [19,20].

## 2. Materials and Methods

The present research was designed as an observational, cross-sectional study, aiming to validate a questionnaire developed to assess dentists’ perceptions regarding the influence of prosthetic restorations on the periodontium. This type of study design was selected for its ability to provide a comprehensive overview of current clinical perspectives at a specific point in time, without manipulating any variables, thus allowing for a realistic capture of naturally occurring professional attitudes and practices.

✓ Study Design and Participant Selection

Data was collected through a self-administered, structured questionnaire, developed by the research team based on current literature and clinical guidelines relevant to prosthodontics and periodontology. The questionnaire was distributed in both printed and electronic formats, ensuring accessibility and flexibility in response collection. Before distribution, the questionnaire underwent face and content validation by a panel of experts in prosthetic and periodontal dentistry to ensure clarity, relevance, and coherence of the items.

The questionnaire was initially administered to a pilot sample of 50 dentists in order to evaluate the clarity and comprehensibility of the items. Based on the feedback received from 39 participants, minor wording adjustments were made to improve item precision and readability. The sample size for the pilot study was pragmatically set at 50 participants, in accordance with methodological recommendations for pilot testing and preliminary scale validation, which indicate that samples between 30 and 50 participants are acceptable for exploratory purposes. Although the recommended ratio of 5–10 participants per item would imply a larger sample for formal validation of this 27-item instrument, this study aimed to conduct an initial psychometric assessment prior to large-scale validation. Therefore, the final sample of 39 completed responses was considered sufficient for the objectives of this exploratory analysis

Participants were recruited through convenience sampling, a non-probabilistic method that allows for the inclusion of respondents based on availability and willingness to participate. This approach, while not aimed at generalizability to the entire population of dentists, is commonly employed in early validation studies to ensure a pragmatic and timely collection of data.

The target population includes licensed dental practitioners currently active in clinical practice. Dentists from various professional backgrounds were invited to participate, including those working in solo private practices, multidisciplinary dental clinics, and public healthcare institutions. Importantly, the inclusion criteria did not impose restrictions based on years of clinical experience, postgraduate training, or specialization, as the intention was to capture a broad and diverse spectrum of opinions and approaches.

Participants were informed about the purpose of the study and the voluntary nature of their participation. Informed consent was obtained prior to questionnaire completion, and confidentiality of responses was ensured in accordance with ethical research standards. No personal identifiers were collected, and all data was anonymized before analysis.

The rationale for selecting a wide range of practitioners was to increase the ecological validity of the questionnaire and test its applicability across various levels of clinical expertise and organizational contexts. By involving both general practitioners and specialists in prosthodontics or periodontology, the study aimed to evaluate whether the instrument is sensitive to differences in clinical knowledge and decision-making processes related to prosthetic–periodontal interactions.

Furthermore, this methodological approach allowed for preliminary insights into current perceptions and practices among Romanian dental professionals, offering a valuable foundation for more extensive future studies—including those using stratified or randomized sampling strategies—to confirm findings and explore professional trends in greater depth.

The data was quantified, and statistical analysis and validation tests were run using descriptive statistics. Microsoft Access was used for parameter-specific analysis, and Excel and the database were used for statistical analysis. Some of the descriptive statistics, as well as Pearson correlations, were utilized in the statistical analysis.

✓ Research Instrument

The questionnaire used in this study was designed to assess dentists’ perceptions and practices regarding the influence of prosthetic restorations on the periodontium. It was structured into three main sections:

General information (age, gender, years of professional experience, specialization).

✓ Section I—Clinical evaluation and attitude toward periodontal health in the context of prosthetic restorations.
✓ Section II—Clinical perceptions and therapeutic decisions related to the prosthesis–periodontium relationship.

Most items were formulated using a 5-point Likert scale, ranging from “Never/Not at all” to “Always/Strongly agree.” Additionally, the questionnaire included closed, semi-open, and one open-ended question to gather the respondent’s personal opinion.

Furthermore, exploratory factor analysis enabled a clear delineation of six conceptual domains, providing insight into the underlying structure of the instrument:

- Factor 1 reflects perceptions regarding the adaptation of fixed prosthetic restorations according to periodontal status, including items on material selection, margin design, and the risk of gingival inflammation.
- Factor 2 is associated with interdisciplinary collaboration and multidisciplinary treatment planning.
- Factor 3 captures aspects related to oral hygiene monitoring and periodontal re-evaluation after treatment.
- Factor 4 expresses core clinical criteria for periodontal health before the placement of definitive restorations.
- Factors 5 and 6 group items related to the impact of removable prostheses and initial clinical assessment, respectively.

✓ Questionnaire Distribution

The questionnaire was distributed in electronic format through digital platforms commonly used by the dental professional community (professional email lists, social media, and online forums for dentists). Participation was anonymous and voluntary, and the estimated time for completion was approximately 5 min.

✓ Questionnaire Validation Process

The content of the questionnaire was evaluated by an expert panel consisting of five experienced dentists specializing in prosthodontics and periodontology. To quantify the level of agreement among the experts, the Content Validity Index (CVI) was employed. Each item was rated on a 4-point scale for clinical relevance and clarity of formulation.

For each item, the Item-level Content Validity Index (I-CVI) was calculated as the proportion of experts who assigned a rating of 3 or 4. All items achieved an I-CVI value of ≥0.80, and the Scale-level CVI (S-CVI/Ave) was calculated to be 0.92, indicating very good content validity of the instrument.

The Item-level Content Validity Index (I-CVI) was calculated as the ratio between the number of experts who rated the item as 3 or 4 and the total number of experts (I-CVI = number of experts scoring 3 or 4/total number of experts).

✓ Reliability (Internal Consistency)

To assess the internal consistency of the scale, Cronbach’s alpha coefficient was calculated for the entire instrument as well as for each thematic section individually (i.e., clinical evaluation and attitude, perceptions and therapeutic decisions). A Cronbach’s alpha value of ≥0.70 was considered acceptable, indicating good internal reliability. In addition, item-total correlations were examined, and the impact of item deletion on the overall alpha value was analyzed to identify potentially redundant or weak items.

✓ Exploratory Factor Analysis (EFA)

To identify the latent structure underlying the measured constructions, Exploratory Factor Analysis (EFA) was conducted using the Principal Component Analysis (PCA) method with Varimax rotation. The adequacy of the data for EFA was evaluated using the Kaiser–Meyer–Olkin (KMO) index with a threshold of >0.60 and Bartlett’s test of sphericity with significance set at *p* < 0.05.

✓ Data Analysis

The collected data was compiled into a central database and subsequently analyzed using SPSS software (version 26.0, IBM, Armonk, NY, USA). Descriptive analysis included frequencies and percentages for categorical responses, while for items measured on a Likert scale, means and standard deviations were calculated.

Depending on the objective of the analysis, comparative statistical tests were applied, such as the Chi-square test, the independent samples *t*-test, and ANOVA (Analysis of Variance). The level of statistical significance was set at *p* < 0.05.

## 3. Results

✓ Questionnaire Validation

The analysis of the internal consistency of the instrument demonstrated an excellent level of reliability. The Cronbach’s alpha coefficient calculated for the entire questionnaire was 0.900, and the standardized item alpha was 0.917, indicating a high degree of item homogeneity and strong response coherence. These values exceed the commonly accepted threshold of 0.70, as recommended in the scientific literature, suggesting that the 27-item instrument consistently measured the intended construct—dentists’ perception of the impact of prosthetic restorations on the periodontium. Therefore, the questionnaire can be considered a reliable tool for use in future research within the field of dental medicine.

The descriptive statistical analysis of the 27 questionnaire items revealed a general trend of strong agreement among dentists with the statements regarding the influence of prosthetic restorations on periodontal health. The mean response values ranged from 2.48 to 4.87 on a 5-point Likert scale, indicating a predominantly positive and responsible perception of the importance of an integrated prosthetic–periodontal approach. These descriptive results highlight that respondents generally acknowledged the importance of integrating periodontal considerations into prosthetic planning.

The highest mean scores were observed for items emphasizing the importance of periodontal health for the success of prosthetic treatment (M = 4.87; SD = 0.34), the need to educate patients on oral hygiene (M = 4.87; SD = 0.34), the negative impact of poor hygiene in removable dentures on gingival tissues (M = 4.82; SD = 0.39), and the recurrence of periodontitis in patients with poorly adapted prosthetic restorations (M = 4.82; SD = 0.45).

In contrast, the lowest scores were recorded for items related to the use of periodontal indices in patient assessment (M = 2.48; SD = 1.19) and the frequency of prosthetic failures caused by insufficient periodontal evaluation (M = 2.77; SD = 0.87), as seen in Table 1.

✓ Item Contribution to Internal Consistency

To evaluate the contribution of each item to the internal consistency of the scale), an analysis of corrected item-total correlations and Cronbach’s alpha if each item was deleted was conducted. Most items showed corrected item-total correlation values above the recommended threshold of 0.40, indicating good internal coherence between the items and the construction being measured. This suggests that the questionnaire items are consistently aligned with the central construct under investigation.

The highest correlations were observed for items referring to the development of multidisciplinary treatment plans (r = 0.733), the selection of prosthetic materials based on periodontal tolerance (r = 0.704), and the integration of periodontology into prosthetic planning (r = 0.704). These findings suggest that these items contribute significantly to the overall consistency of the scale.

On the other hand, some items recorded low correlations, falling below the minimum recommended threshold (r < 0.30), such as the item regarding abutment tooth mobility in removable partial dentures (r = 0.006) and the item related to excluding patients with active periodontitis (r = 0.196). While the removal of these items would lead to a slight increase in the overall Cronbach’s alpha value (up to 0.907 and 0.905, respectively), the improvement is marginal and does not justify their exclusion without further conceptual analysis.

Additionally, the overall Cronbach’s alpha remained stable or decreased slightly with the deletion of most items, supporting the notion that each contributes meaningfully to the reliability of the scale. This result confirms that the instrument reliably captures dentists’ perceptions and can support future clinical and educational assessments.

Therefore, the results support the conceptual homogeneity of the questionnaire and confirm that the included items consistently measure dentists’ perceptions regarding the interaction between prosthetic restorations and the periodontium, without requiring major structural modifications to the instrument, as can be seen in Table 2 below:

✓ Intraclass Correlation Coefficient (ICC) Analysis

The internal consistency of the scale was further evaluated by calculating the Intraclass Correlation Coefficient (ICC) using the two-way mixed model with average measures under the assumption of absolute agreement among items (Table 3). The ICC value for average measures was 0.900, with a 95% confidence interval ranging from 0.849 to 0.940, indicating an excellent level of consistency among the items. Overall, these ICC results further reinforce the consistency and reproducibility of the instrument, supporting its applicability in diverse dental practice contexts.

In contrast, the ICC value for single measures was 0.250, suggesting a higher degree of variability when items are considered individually. However, this variability is compensated when item scores are aggregated, reinforcing the reliability of the overall scale.

The associated F-test (F = 9.996, *p* < 0.001) was statistically significant, confirming that the variability between items was substantially lower than would be expected by chance. These ICC results are consistent with the previously reported high Cronbach’s alpha values and provide robust support for the internal reliability and reproducibility of the scale, thereby validating the questionnaire for future research on dentists’ perceptions regarding the interaction between prosthetic restorations and periodontal health, as can be seen in Table 3.

✓ Communalities Analysis

The communalities values reflect the proportion of each item’s variance that is explained by the identified factor structure. In this analysis, the extraction values (post-rotation) were high for most items, ranging between 0.549 and 0.966, indicating a good representation of each item within the factorial model. (Table 3) The highest communality values were recorded for the items “The periodontal condition influences the choice of prosthetic restoration type” and “Fixed prosthetic restorations can promote gingival inflammation if not properly adapted” (both with an extraction value of 0.966), as well as for “Patients must have a stable periodontium before definitive restoration placement“ and “A subgingival prosthetic margin is a risk factor for periodontal damage” (both with 0.938).

These high values confirm that these items contribute significantly to the definition of the latent construction and are well correlated with the extracted factors. Conversely, the lowest communality was observed for the item “I have encountered prosthetic failures caused by insufficient periodontal evaluation” (0.549), which, although below the ideal threshold of 0.60, still falls within the acceptable range for exploratory research.

Overall, the communalities support the theoretical adequacy of the instrument, indicating that the majority of items are well represented by the extracted factors and that the model explains a substantial proportion of the variance in responses (Table 4).

✓ Factor Structure and Construct Validity

The factorial model obtained through Exploratory Factor Analysis (EFA) reveals a clear, coherent, and theoretically justified structure, in which items logically group into relevant conceptual dimensions: clinical evaluation, therapeutic decision-making, interdisciplinary collaboration, and the impact of fixed or removable restorations on the periodontium. The high communalities, in combination with distinct factor loadings and the previously reported adequacy indices (KMO and Bartlett’s test), provide strong support for the construct validity of the questionnaire.

The EFA identified a solution consisting of eight principal factors, each with an eigenvalue greater than 1, in accordance with Kaiser’s criterion. These eight factors were retained following Principal Component Analysis (PCA) and together accounted for 81.68% of the total variance, a high percentage that supports the complexity and thematic coverage of the instrument.

In its initial form, the first factor exhibited an eigenvalue of 9.695, explaining 35.91% of the total variance, suggesting the presence of a dominant latent dimension in respondents’ attitudes and perceptions. After applying Varimax rotation, the variance was more evenly distributed across factors: Factor 1 retained 17.02% of the variance, while the remaining factors contributed between 5.5% and 12.8%, resulting in a clearer and more interpretable factor structure.

Cumulatively, the first five rotated factors accounted for approximately 63.25% of the total variance, indicating that a significant portion of item variability can be attributed to these essential latent dimensions: clinical assessment, therapeutic decision-making, hygiene and monitoring, interdisciplinary collaboration, and the impact of removable restorations. This robust factorial solution is further supported by the high communalities, strong KMO index, and the significance of Bartlett’s test, all of which confirm the construct validity of the scale.

The results suggest that the questionnaire consists of a well-defined factorial structure with eight meaningful dimensions, adequately reflecting the complexity of dentists’ perceptions regarding the relationship between prosthetic treatments and periodontal health. The high percentage of explained variance and the consistency of items within each factor validate the instrument from a psychometric perspective, offering a solid foundation for its use in future research and educational interventions as mentioned in Table 5.

✓ Exploratory Factor Analysis and Construct Validation

To validate the latent structure of the questionnaire designed to assess dentists’ perceptions regarding the influence of prosthetic restorations on the periodontium, an Exploratory Factor Analysis (EFA) was conducted using the Principal Component Analysis (PCA) extraction method with Varimax orthogonal rotation. The suitability of the data for factor analysis was confirmed by the Kaiser–Meyer–Olkin (KMO) index and Bartlett’s test of sphericity, both of which indicated sufficient inter-item correlations.

The results revealed the presence of eight principal factors with eigenvalues greater than 1, which together explained 81.68% of the total variance. This high proportion of explained variance suggests a robust factorial structure, relevant for a multidimensional investigation of dentists’ clinical perceptions and attitudes. After rotation, the variance was more evenly distributed across the factors, with individual contributions ranging from 5.5% to 17%, facilitating a clearer interpretation of each latent dimension.

The final two factors reflect clinical decision-making regarding the exclusion of patients with active periodontitis and experiences with prosthetic failures, offering insight into periodontal management in prosthetic practice.

The post-extraction communalities ranged from 0.549 to 0.966, indicating that the items are well represented by the factorial structure. The most strongly represented items refer to the adaptation of fixed restorations and the integration of periodontology into prosthetic practice, while the least represented item was the one concerning prosthetic failures related to insufficient periodontal evaluation, which, despite its lower value, still falls within the acceptable range for exploratory studies.

The exploratory factor analysis confirms that the questionnaire possesses a clear and coherent multidimensional structure, consisting of eight theoretically and clinically relevant dimensions. The combination of strong factor loadings, high total explained variance, and solid communalities supports the construct validity of the instrument, validating it as a reliable and psychometrically sound tool for assessing dental professionals’ perceptions regarding the relationship between prosthetic restorations and periodontal health. Together, these findings demonstrate that the factorial structure effectively reflects the multidimensional nature of dentists’ perceptions regarding prosthetic–periodontal interactions.

## 4. Discussion

The current study aimed to develop and validate a questionnaire addressing the perceptions and attitudes of dental professionals regarding the interrelationship between prosthetic treatments and periodontal health. In the present study, the indicators provide preliminary evidence supporting the psychometric adequacy of the structure, within the limitations of the design and sample. The Cronbach’s alpha coefficient of 0.900 exceeds the commonly accepted threshold of 0.7, indicating excellent reliability [21]. These results provide initial evidence of validity and internal consistency, within the exploratory limitations of this pilot study.

A limitation is that only 39 participants completed the pilot survey, which is slightly less than the targeted 50. However, given the strong psychometric results—high internal consistency, suitable factor structure, and adequate sampling adequacy—this sample size was sufficient for exploratory validation, although confirmatory studies with larger samples are warranted.

Through exploratory factor analysis (EFA), eight distinct factors emerged, explaining over 81% of the total variance. This level of explained variance is indicative of a well-structured tool, particularly in social and clinical sciences, where a threshold of 60% is often considered satisfactory [17]. The resulting factor structure supports the theoretical framework underpinning the design of the questionnaire and is consistent with previous studies that developed tools in interdisciplinary areas such as prosthodontics, periodontology, and oral health behavior [18]. The final sample size of 39 participants, while sufficient for an initial exploratory assessment, remains small for robust psychometric validation, particularly for exploratory factor analysis. Additionally, the convenience sampling approach may have introduced selection bias, as individuals with a particular interest in periodontal–prosthetic integration may have been more likely to participate, potentially resulting in higher-than-average perception scores and overestimating certain psychometric indicators. However, these results should be interpreted as preliminary and hypothesis-generating, providing a foundation for future research using larger, random, or stratified samples to confirm and extend these findings.

From the perspective of content validity, the questionnaire captures essential domains related to periodontal–prosthetic integration: biological width, crown contouring, marginal adaptation, maintenance of hygiene, and failure prevention strategies. These domains reflect current guidelines and consensus papers emphasizing the need for synergy between prosthetic design and periodontal preservation [22,23].

The high average agreement among respondents regarding the importance of respecting the periodontal biotype, ensuring proper emergence profiles, and maintaining a cleansable restoration surface suggests that Romanian dentists exhibit a high level of theoretical understanding. This is aligned with findings from international surveys, which show that clinicians are increasingly aware of the importance of soft-tissue management in prosthodontic planning [24].

However, several areas in the questionnaire revealed relatively lower scores, including the routine use of periodontal indices in prosthetic planning or the implementation of regular periodontal re-evaluation post-restoration. These gaps point toward a disconnect between theoretical knowledge and clinical practice—a challenge also documented in the literature. These findings may suggest a less frequent application of standardized periodontal diagnostic tools in routine dental practice. The relatively low standard deviations, mostly below 1.0, reflect a high degree of response homogeneity, supporting the consistency of perceptions among respondents and reinforcing the relevance of the topic within current dental practice.

Studies have shown that while dental professionals acknowledge the relevance of periodontal considerations, these are not always translated into consistent clinical workflows [17].

Another important finding is the variation in responses related to the management of prosthetic failures. Although most practitioners recognize the role of plaque-induced inflammation and poor marginal fit in treatment failure, fewer appear to routinely trace such failures back to insufficient periodontal evaluation or pre-prosthetic therapy. This reflects the need for continuing education and clinical protocols that reinforce interdisciplinary treatment planning [25].

In terms of practical application, the validated questionnaire offers a structured instrument that can serve multiple functions. It can be employed in academic settings to evaluate knowledge and attitudes among students and residents, in clinical audits to assess practice patterns, and in research studies to explore factors influencing treatment outcomes. The tool may also assist in benchmarking periodontal awareness among prosthodontists and general practitioners alike.

Despite its strengths, this study presents certain limitations. The sample size, though adequate for exploration validation, limits the generalizability of the findings. Future research could explore cross-cultural validation and comparison among subgroups (e.g., by years of experience or type of dental training). Additionally, a Confirmatory Factor Analysis (CFA) in a separate sample would further validate the dimensional structure identified in this study.

The relatively small sample size of 39 participants represents a limitation of this study, particularly with respect to the robustness of the psychometric validation and generalizability of the findings. The use of convenience sampling further limits representativeness and may have introduced selection bias, potentially leading to overestimation of reliability and validity metrics if dentists with a greater interest in periodontal–prosthetic integration were more likely to participate. While a pilot sample of 50 participants was initially targeted, only 39 complete responses were valid to be included in the current study. Future studies should aim to improve the applicability of this questionnaire in larger, randomly selected, or stratified samples—e.g., stratified by specialization, practice setting, or years of professional experience—to confirm and extend the present findings.

While the sample size is modest, we emphasize that the statistical indicators (e.g., high communalities, strong KMO and Bartlett’s tests, and clear factor structure explaining over 81% of variance) provide robust evidence supporting the validity and reliability of the instrument. Therefore, we consider these results to represent a solid initial validation, appropriate for exploratory purposes, while acknowledging that future studies in larger samples could further strengthen generalizability.

The descriptive results of this study yield valuable insights into current clinical practices and perceptions among Romanian dentists regarding the relationship between periodontal health and prosthetic rehabilitation. The high mean scores observed for items related to the theoretical principles of periodontal–prosthetic integration—such as respecting the biological width, emergence profile, and the design of restorations to allow for adequate hygiene—suggest a commendable level of awareness and knowledge of interdisciplinary best practices. However, this apparent knowledge is not fully reflected in self-reported behaviors, as evidenced by the comparatively low scores regarding the routine use of periodontal indices and the attribution of prosthetic failures to inadequate periodontal evaluation prior to treatment.

These findings highlight critical practice gaps that may compromise the long-term success of prosthetic treatments and periodontal stability in patients.

The underuse of standardized periodontal indices in prosthetic planning suggests that periodontal diagnostic tools are not sufficiently integrated into routine workflows, potentially limiting the early detection and management of periodontal conditions that could affect restorative outcomes. Similarly, the limited recognition of prosthetic failures as being linked to insufficient periodontal evaluation reflects a need to strengthen clinical reasoning and interdisciplinary diagnostic approaches among practitioners.

Addressing these deficiencies requires a dual strategy: enhancing the dental curriculum to ensure that students develop not only theoretical knowledge but also practical competencies in periodontal assessment within prosthetic treatment planning and providing continuing education programs for practicing dentists to reinforce the importance of standardized periodontal diagnostics and interdisciplinary management in daily practice. Developing and promoting clinical protocols that integrate periodontal assessments as a standard component of prosthetic workflows may facilitate a better translation of knowledge into practice.

These observations align closely with the stated aim of this study: to generate evidence that can inform curriculum reform and clinical protocol development, ultimately contributing to improved standards of care and interdisciplinary collaboration in Romanian dental clinical practice.

## 5. Conclusions

This study demonstrated that Romanian dentists hold a predominantly positive perception regarding the influence of prosthetic restorations on periodontal health, emphasizing the critical role of periodontal integrity in the success of prosthetic treatments.

The instrument used underwent an exploratory validation process, which indicated good internal consistency and provided initial evidence supporting the underlying psychometric structure. Construct validity was supported by the high communalities and a strong KMO coefficient, indicating that the items were well represented and the underlying theoretical structure was solid.

The statistical findings obtained in this study suggest the preliminary psychometric adequacy of the scale, particularly regarding construct validity and internal consistency, within the limits of an exploratory design and a non-probabilistic sample.

In conclusion, this study contributes to the advancement of interdisciplinary oral healthcare by providing a validated tool for assessing perceptions at the interface of periodontics and prosthodontics. By identifying current strengths and gaps in practice, it supports the broader goal of promoting biologically driven, patient-centered prosthetic rehabilitation.

## Figures and Tables

**Table 1:** Descriptive analysis of the 27 questionnaire items.

Item	Mean	Standard Deviation	N
I assess the periodontal status of each abutment tooth before starting prosthetic treatment	4.56	0.6	39
I collaborate with a periodontist in complex prosthetic cases	3.67	1.42	39
I consider periodontal health a mandatory condition for the success of a prosthetic restoration	4.87	0.34	39
I perform periodontal treatment (scaling, root planing) before prosthetic restoration	4.62	0.85	39
I educate patients on oral hygiene as part of the prosthetic plan	4.87	0.34	39
I use periodontal indices (e.g., gingival index, CPITN) when assessing patients indicated for prosthetic treatment	2.49	1.19	39
I exclude patients with active periodontitis from the prosthetic treatment plan	3.44	1.29	39
I use provisional restorations to assess periodontal tolerance before finalizing treatment	3.64	1.06	39
I have encountered prosthetic failures caused by insufficient periodontal evaluation	2.77	0.87	39
I monitor gingival status over time after cementing definitive restorations	4.03	0.78	39
I consider correct occlusal guidance essential for protecting the periodontium in prosthetic restoration	4.46	0.82	39
The periodontal condition influences the choice of prosthetic restoration type	4.41	0.75	39
Patients must have a stable periodontium before definitive restoration placement	4.77	0.43	39
I consider the integration of periodontology into prosthetics a mandatory standard in modern practice	4.82	0.45	39
I select materials and design for prosthetic restorations based on periodontal tolerance	4.21	0.77	39
I conduct periodic clinical re-evaluations for patients with prosthetic restorations (fixed or removable)	4.13	0.92	39
I develop multidisciplinary treatment plans (prosthetics + periodontology) when needed	4.26	0.97	39
Fixed prosthetic restorations can promote gingival inflammation if not properly adapted	4.41	0.75	39
A subgingival prosthetic margin is a risk factor for periodontal damage	4.77	0.43	39
I have observed recurrence of periodontitis in patients with old or poorly adapted prosthetic restorations	4.82	0.45	39
Improperly adapted fixed restorations can lead to gingival recession	4.21	0.77	39
Overcontoured crowns impair hygiene and promote gingival inflammation	4.13	0.92	39
Removable prostheses can contribute to bone resorption in support areas	4.26	0.97	39
The stability of removable prostheses affects the periodontal health of remaining teeth	4.62	0.63	39
Abutment teeth for removable partial dentures tend to develop mobility over time	4.08	0.77	39
Clasp design can negatively influence the mobility and periodontium of abutment teeth	4.26	0.85	39
Poor hygiene of removable prostheses contributes to gingival inflammation	4.82	0.39	39

**Table 2:** Analysis of “Cronbach’s alpha if item deleted” and corrected item-total correlation.

Item	Scale Mean if Item Deleted	Scale Variance if Item Deleted	Corrected Item-Total Correlation	Cronbach’s Alpha if Item Deleted
I assess the periodontal status of each abutment tooth before starting prosthetic treatment	109.7949	132.588	0.201	0.901
I collaborate with a periodontist in complex prosthetic cases	110.6923	118.798	0.481	0.899
I consider periodontal health a mandatory condition for the success of a prosthetic restoration	109.4872	132.151	0.442	0.898
I perform periodontal treatment (scaling, root planing) before prosthetic restoration	109.7436	123.09	0.634	0.893
I educate patients on oral hygiene as part of the prosthetic plan	109.4872	131.835	0.483	0.898
I use periodontal indices (e.g., gingival index, CPITN) for evaluating patients indicated for prosthetic treatment	111.8718	122.746	0.438	0.899
I exclude patients with active periodontitis from the prosthetic treatment plan	110.9231	128.283	0.196	0.907
I use provisional restorations to assess periodontal tolerance before finalizing treatment	110.7179	121.997	0.536	0.895
I have encountered prosthetic failures caused by insufficient periodontal evaluation	111.5897	130.564	0.22	0.902
I monitor gingival status over time after cementing definitive restorations	110.3333	123.754	0.656	0.893
I consider correct occlusal guidance essential for protecting the periodontium in prosthetic restoration	109.8974	125.673	0.508	0.896
The periodontal condition influences the choice of prosthetic restoration type	109.9487	123.576	0.693	0.892
Patients must have a stable periodontium before definitive restoration placement	109.5897	131.143	0.449	0.898
I consider the integration of periodontology into prosthetics a mandatory standard in modern practice	109.5385	128.308	0.704	0.895
I select materials and design for prosthetic restorations based on periodontal tolerance	110.1538	123.134	0.704	0.892
I conduct periodic clinical re-evaluations for patients with prosthetic restorations (fixed or removable)	110.2308	120.866	0.69	0.892
I develop multidisciplinary treatment plans (prosthetics + periodontology) when needed	110.1026	119.305	0.733	0.89
Fixed prosthetic restorations can promote gingival inflammation if not properly adapted	109.9487	123.576	0.693	0.892
A subgingival prosthetic margin is a risk factor for periodontal damage	109.5897	131.143	0.449	0.898
I have observed recurrence of periodontitis in patients with old or poorly adapted prosthetic restorations	109.5385	128.308	0.704	0.895
Improperly adapted fixed restorations can lead to gingival recession	110.1538	123.134	0.704	0.892
Overcontoured crowns impair hygiene and promote gingival inflammation	110.2308	120.866	0.69	0.892
Removable prostheses can contribute to bone resorption in support areas	110.1026	119.305	0.733	0.89
The stability of removable prostheses affects the periodontal health of remaining teeth	109.7436	129.196	0.425	0.898
Abutment teeth for removable partial dentures tend to develop mobility over time	110.2821	134.997	0.006	0.905
Clasp design can negatively influence the mobility and periodontium of abutment teeth	110.1026	129.516	0.283	0.9
Poor hygiene of removable prostheses contributes to gingival inflammation	109.5385	132.992	0.286	0.9

**Table 3:** Intraclass correlation coefficient.

	Intraclass Correlation	95% Confidence Interval		F-Test with True Value 0			
		Lower Bound	Upper Bound	Value	df1	df2	Sig
Single measures	0.250	0.172	0.368	9.996	38	988	0.000
Average measures	0.900	0.849	0.940	9.996	38	988	0.000

**Table 4:** Communalities analysis.

Item	Initial	Extraction
I assess the periodontal status of each abutment tooth before starting prosthetic treatment	1.0	0.783
I collaborate with a periodontist in complex prosthetic cases	1.0	0.809
I consider periodontal health a mandatory condition for the success of a prosthetic restoration	1.0	0.729
I perform periodontal treatment (scaling, root planing) before prosthetic restoration	1.0	0.758
I educate patients on oral hygiene as part of the prosthetic plan	1.0	0.717
I use periodontal indices (e.g., gingival index, CPITN) for evaluating patients indicated for prosthetic treatment	1.0	0.566
I exclude patients with active periodontitis from the prosthetic treatment plan	1.0	0.817
I use provisional restorations to assess periodontal tolerance before finalizing treatment	1.0	0.8
I have encountered prosthetic failures caused by insufficient periodontal evaluation	1.0	0.549
I monitor gingival status over time after cementing definitive restorations	1.0	0.784
I consider correct occlusal guidance essential for protecting the periodontium in prosthetic restoration	1.0	0.748
The periodontal condition influences the choice of prosthetic restoration type	1.0	0.966
Patients must have a stable periodontium before definitive restoration placement	1.0	0.938
I consider the integration of periodontology into prosthetics a mandatory standard in modern practice	1.0	0.875
I select materials and design for prosthetic restorations based on periodontal tolerance	1.0	0.906
I conduct periodic clinical re-evaluations for patients with prosthetic restorations (fixed or removable)	1.0	0.877
I develop multidisciplinary treatment plans (prosthetics + periodontology) when needed	1.0	0.887
Fixed prosthetic restorations can promote gingival inflammation if not properly adapted	1.0	0.966
A subgingival prosthetic margin is a risk factor for periodontal damage	1.0	0.938
I have observed recurrence of periodontitis in patients with old or poorly adapted prosthetic restorations	1.0	0.875
Improperly adapted fixed restorations can lead to gingival recession	1.0	0.906
Overcontoured crowns impair hygiene and promote gingival inflammation	1.0	0.877
Removable prostheses can contribute to bone resorption in support areas	1.0	0.887
The stability of removable prostheses affects the periodontal health of remaining teeth	1.0	0.837
Abutment teeth for removable partial dentures tend to develop mobility over time	1.0	0.778
Clasp design can negatively influence the mobility and periodontium of abutment teeth	1.0	0.8
Poor hygiene of removable prostheses contributes to gingival inflammation	1.0	0.684

**Table 5:** Total variance.

Component	Initial Eigenvalues			Extraction Sums of Squared Loadings			Rotation Sums of Squared Loadings		
	Total	% of Variance	Cumulative %	Total	% of Variance	Cumulative %	Total	% of Variance	Cumulative %
1	9.695	35.909	35.909	9.695	35.909	35.909	4.596	17.024	17.024
2	2.999	11.107	47.017	2.999	11.107	47.017	3.471	12.854	29.878
3	2.177	8.064	55.081	2.177	8.064	55.081	3.265	12.092	41.970
4	1.808	6.696	61.777	1.808	6.696	61.777	2.878	10.659	52.628
5	1.611	5.966	67.743	1.611	5.966	67.743	2.868	10.621	63.249
6	1.366	5.061	72.804	1.366	5.061	72.804	1.831	6.780	70.029
7	1.269	4.699	77.503	1.269	4.699	77.503	1.658	6.139	76.168
8	1.127	4.174	81.678	1.127	4.174	81.678	1.488	5.510	81.678

Extraction method: principal component analysis.

## Data Availability

All data are available from the corresponding author upon reasonable request.

## Abbreviations

The following abbreviations are used in this manuscript:

CVI	Content Validity Index
EFA	Exploratory Factor Analysis
PCA	Principal Component Analysis
KMO	Kaiser–Meyer–Olkin
I-CVI	Item-level Content Validity Index
ICC	Intraclass Correlation Coefficient
